# Judo across ages: coaches’ perspectives on key elements of intergenerational training programmes

**DOI:** 10.3389/fpsyg.2025.1678557

**Published:** 2025-11-12

**Authors:** Andrea Perazzetti, Flavia Guidotti, Laura Capranica, Envic Galea, Tibor Kozsla, Attilio Sacripanti, Nuša Lampe, Špela Lampe, Iris Spanjol, Toma Jelušić, Slavisa Bradic, Maria-Loredana Lascau, Alina Rodica-Borza, Raúl Camacho Pérez, Fernando Diéguez Rodríguez-Montero, Mesut Kapan, Kaya Gezeker, Katja Rudas, Mojca Doupona, Angela Magnanini, Simone Ciaccioni

**Affiliations:** 1 Department of Movement, Human and Health Sciences, University of Rome “Foro Italico”, Rome, Italy; 2 Department of Human Sciences and Promotion of Quality of Life, University of Rome "San Raffaele", Rome, Italy; 3 International Judo Federation Academy Foundation, Malta, Malta; 4 Judo Club Golovec, Ljubljana, Slovenia; 5 Zajednica Sportskih Udruga Grada Rijeke “Riječki Sportski Savez”, Rijeka, Croatia; 6 Judo Club Liberty Oradea, Oradea, Romania; 7 Club de Judo Newton, Sevilla La Nueva, Spain; 8 Izmir Alsancak Gymnastics Specialized Sports Club, Izmir, Türkiye; 9 European Judo Union, Vienna, Austria; 10 Faculty of Sport, University of Ljubljana, Ljubljana, Slovenia; 11 Department of Education and Sport Sciences, Pegaso Telematic University, Naples, Italy

**Keywords:** sustainable education, martial arts, combat sports, judo, older adults, wellbeing, intergenerational sport

## Abstract

**Introduction:**

Judo is an Olympic combat sport and martial art known for promoting physical, psychological, and social wellbeing across all ages. In line with European initiatives encouraging intergenerational activities, judo presents a valuable opportunity to connect younger and older individuals through shared physical activities. Conducted within the framework of the ERASMUS+ Sport JOY Project, this study explored judo coaches’ knowledge, opinions, and experiences to identify key components for designing effective and sustainable intergenerational judo programmes.

**Method:**

A survey was distributed to 349 judo coaches (*M* = 82% and *F* = 18%) affiliated with international judo organisations and clubs to investigate their views on key characteristics, roles, barriers, and facilitators of intergenerational judo, as well as their coaching philosophies and educational needs.

**Results:**

Main findings revealed that in such programmes coaches prioritize safety and skill ex-change while promoting social interactions. Significant differences emerged based on coaches’ demographics, experience, and education level, particularly regarding their perspectives on coaching roles, communication challenges, and motivational aspects.

**Discussion:**

From a psychological perspective, these programmes foster mutual respect, empathy, and intergenerational understanding, contributing to enhanced mental wellbeing and a stronger sense of belonging among participants. Indeed, the study provides essential insights to inform the creation of inclusive, engaging, and sustainable training strategies that promote active ageing, mutual learning, and social cohesion through judo.

## 1 Introduction

As declared by the European Commission in 2024, Europe must support both young and old citizens by recognizing their commonalities and embracing their diversity, from their cultural heritage to sport at all levels (European Commission and Micallef, 2024). Whilst solidarity between generations is enshrined in European treaties, the decisions we take today have long-term consequences for the next generations to come. In this context, intergenerational sports programmes represent a promising strategy to stimulate active ageing, strengthen social connections, and link communities with the overarching goal of fostering a healthy and more cohesive society (European Commission, Directorate-General for Education, Youth, Sport and Culture, 2020). Through reciprocal care and shared activities, these intergenerational interactions make older people feel useful by assisting younger generations and maintaining their optimism and vitality (Chiosso, 2012). Particularly through shared sport activities and common learning experiences, each generation can benefit from the knowledge of the others, finding a balance between mutual support and independence (Kolb, 2014).

Among the wide range of sport disciplines, judo, an Olympic sport and traditional Japanese martial art, stands out as a valuable medium for intergenerational engagement. Judo is renowned for promoting both mental and physical health (Drid et al., 2021; Kowalczyk et al., 2023; Palumbo et al., 2023) and it simultaneously serves as a means of cultural transmission and intergenerational human growth, grounded in the traditions of discipline, respect, and physical ability (Kano, 2013). Its inclusive nature appeals to practitioners of all ages, offering diverse benefits such as better functional abilities (Arkkukangas et al., 2021; Odaka et al., 2023; Jadczak et al., 2024), positive anthropometric and walking proficiency (Ciaccioni et al., 2019; Ciaccioni et al., 2020) and improved psychological health (Muiños and Ballesteros, 2015; DelCastillo-Andrés et al., 2018; Kujach et al., 2022). To ensure judo continues to evolve as a lifelong practice, it is crucial to understand how it can foster relationships across different age groups, facilitate the sharing of knowledge, and uphold its core principles.

Building on these premises, the European ERASMUS+Sport EDJCO Project was developed to design and validate an evidence and eminence-based online and self-regulated educational programme tailored for judo coaches for training older practitioners. The project provided comprehensive modules encompassing both theoretical and practical strategies for effective communication, ageing process understanding, and judo techniques adaptation to create inclusive and engaging training conditions (Ciaccioni et al., 2024a). However, current research on the potential of judo as an intergenerational sport activity is in its early development stage. Such activities could provide a valuable framework where experienced practitioners share their knowledge, while younger generations bring fresh perspectives, energy, and adaptability. Building on the judo fundamentals and its core principles of maximum efficiency (Seiryoku-Zenyo) and mutual prosperity (Jita-Kyoei) (Kano, 2013), a recent umbrella review (Ciaccioni et al., 2024b) outlined the key guidelines for intergenerational judo activities, providing an extensive analysis of programmes’ main characteristics, barriers, and facilitators, and highlighting relevant judo coaches’ roles and responsibilities (Table 1).

**Table 1:** Overview of guidelines for intergenerational judo activities (Adapted from Ciaccioni et al., 2024b).

Key characteristics	Judo coach’s role	Barriers	Facilitators
Adaptability	Adaptability	Age differences	Adapted teaching and learning styles
Collaboration	Community	Different learning styles and preferences	Inclusive training sessions
Inclusion	Facilitator	Environmental factors	Intergenerational mentorship
Safety	Motivator	Generational stereotypes and prejudices	Mutual respect and empathy
Skill exchange	Role model	Infrastructure challenges	Recognition & celebration of achievements
Social interactions	Safety supervisor	Lack of motivation and interest	Shared passion for sport and judo
Variety	Technical expert	Language and communication barriers	Social interactions
		Physical limitations	Supportive community and facilities

Given judo’s potential to foster constructive social change through intergenerational interactions rooted in respect and empathy, an urging need emerged for tailored programmes with specific coach training to address intergenerational contexts, which remain unexplored despite existing online courses by the International Judo Federation Academy (IJFA). In response to this call, the new ERASMUS+Sport collaborative partnership ‘Judo connecting Older and Younger generations’ (JOY) Project encompasses international partners from academic institutions and high-level sport organisations. Its goal is to enhance the participation of young and older individuals in intergenerational judo activities for their holistic development and successful ageing, respectively. Within this framework, the present study aimed to gather the opinions, knowledge, and experiences of judo coaches regarding the most relevant aspects to be included in an intergenerational training programme. By examining coaching philosophies, and diverse backgrounds of coaches working in intergenerational judo, this research sought to identify key practices, learning needs, and educational strategies that facilitate knowledge transfer and cooperation between different generations within the judo community.

## 2 Materials and methods

This study was performed under the ERASMUS+Sport collaborative partnership ‘Judo connecting Older and Younger generations’ (JOY) Project (ERASMUS-SPORT-2023-SCP; number: 101133628), coordinated by the Croatian Judo Klub Rijeka and supported and supervised by the University of Rome ‘Foro Italico’. All data collected through the survey were used anonymously. This study ensures stringent rigor, meaningful coherence, and resonance of results by adhering to excellent research guidelines for methodological procedures on data acquisition and analysis with ethical compliance conforming to the Declaration of Helsinki (Boateng et al., 2018; Rose, 2019), approved by the IRB of the University of Rome ‘Foro Italico’ (CAR 202/2024).

### 2.1 Study design

After the authors elaborated on the survey’s first draft, a review on its clarity, format, and content was requested to the whole JOY team constituted by two Universities (Rome ‘Foro Italico’, Italy, and Ljubljana, Slovenia), five high-level judo clubs (Croatian JK Rijeka, Turkish IAGC, Slovenian JK Golovec, Romanian JC Liberty, and Spanish JC Newton), two international sport organisations, of which the International Judo Federation Academy-IJFA, and the European Judo Federation, which included judo referees, coaches, managers and professors with certified qualifications and long-term experience.

The final version of the survey encompassed 21 closed-ended questions and 1 open-short question, divided into six sections as follows: (i) Socio-demographic characteristics (13 questions); (ii) Key characteristics (2 questions); (iii) Judo coach’s role (2 questions); (iv) Barriers (2 questions), (v) Facilitators (2 questions), and a (vi) Final open question (1 question). In particular:

- Socio-demographic characteristics (Section 1): General socio-demographic information requests about the coaches, including gender, age, dan level, experience as an elite judoka and years of coaching. Moreover, it investigated trained groups’ age categories, the coach’s involvement in intergenerational activities, philosophy, beliefs, and ways for continuing learning (Perazzetti et al., 2023).
- Key characteristics (Section 2): Coaches were requested to evaluate key features for an educational programme on intergenerational judo, encompassing adaptability, collaboration, inclusion, safety, skill exchange, social interactions, and variety. They assessed the perceived relevance and relevance of these characteristics on a t-point Likert scale.
- Judo coach’s roles (Section 3): Coaches were requested to assess and rank (Likert scale 1–7) their perceived relevance of the main coach’s role in intergenerational activities, such as adaptability specialist, community builder, facilitator, motivator, role model, safety supervisor, and technical expert.
- Barriers (Section 4): Coaches were requested to assess (Likert scale 1–7) and rank (scale 1–8) their perceived impact of obstacles to intergenerational activities, including age differences, learning styles, environmental factors, generational stereotypes, infrastructure challenges, lack of motivation, language barriers, and physical limitations.
- Facilitators (Section 5): Coaches were requested to assess (Likert scale 1–7) and rank (scale 1–8) their perceived impact of the factors that support intergenerational activities, such as adapted teaching styles, inclusive training sessions, intergenerational mentorship, mutual respect and empathy, recognition of achievements, shared passion, social interactions, and a supportive community.
- Final open question (Section 6): Coaches were requested to provide insights or suggestions on additional elements that should be considered in an educational programme for intergenerational judo coaching.

The questionnaire was built in digital format through the Google Forms platform (Perazzetti et al., 2023). Each item was explained in depth by means of the PubMed definition or the respective terminology used in the National Language Medical controlled vocabulary thesaurus (S1). To avoid missing data, the closed-ended questions required compulsory answers.

To ensure accuracy and relevance of the outcomes, the JOY research team developed, translated and distributed the survey to judo coaches included in the IJFA mailing lists.

### 2.2 Participants

All the participants surveyed in this study (*n* = 349) were judo coaches involved in the 2023/2024 and 2024/2025 sport seasons of their respective judo clubs. The questionnaire was sent to 2,178 coaches, of whom 349 responded (response rate of 16.02%). Notably, all 349 responses analysed were unique and complete, as the survey platform automatically prevented duplication or incomplete submissions.

### 2.3 Data analysis

**Figure 1:**
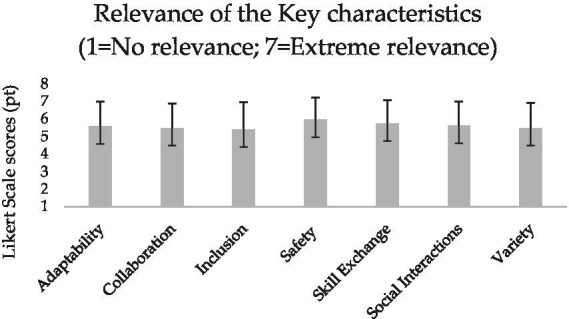
Mean and standard deviation of the coaches’ perceived relevance of the key characteristics of intergenerational judo.

**Figure 2:**
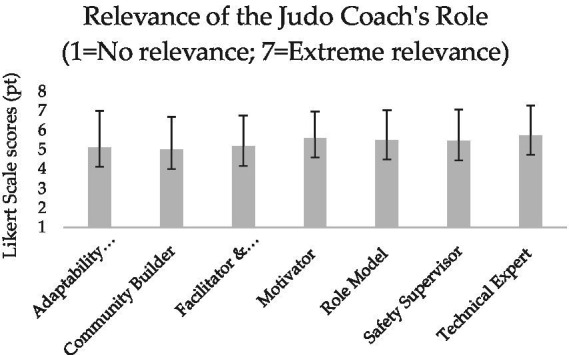
Mean and standard deviation of the coaches’ perceived key role of intergenerational judo.

**Figure 3:**
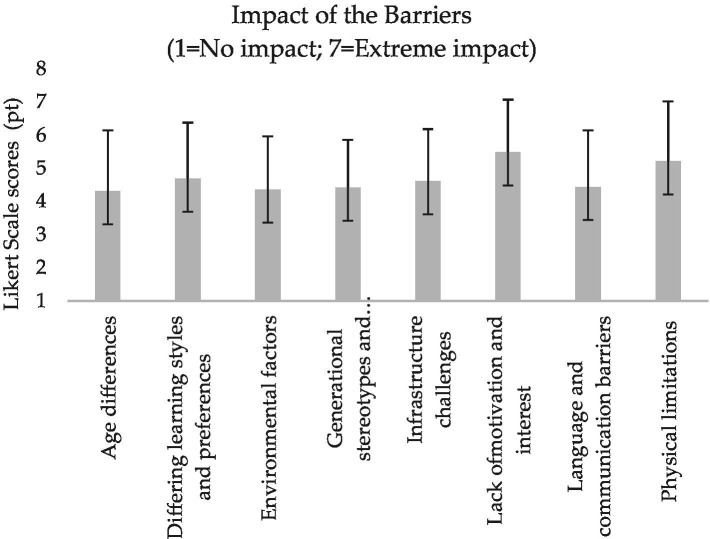
Mean and standard deviation of the coaches’ perceived facilitators of intergenerational judo.

**Figure 4:**
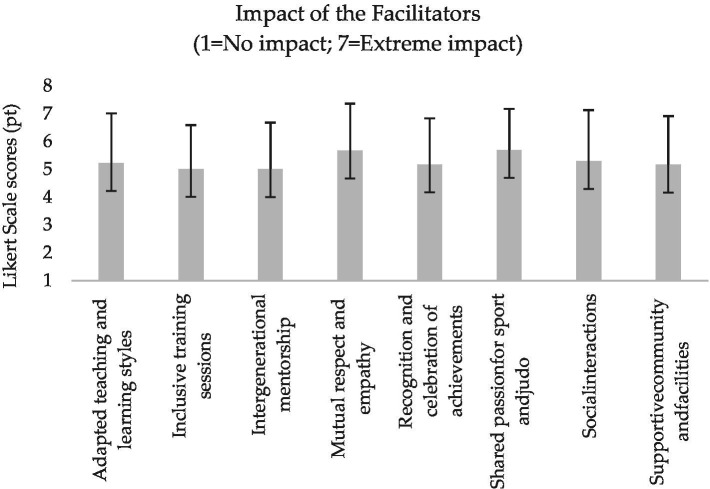
Mean and standard deviation of the coaches’ perceived barriers of intergenerational judo.

**Figure 5:**
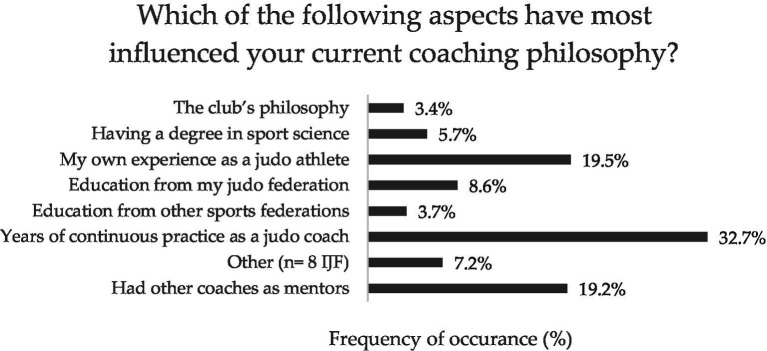
Answers’ percentages of the coaches’ perceived most influencing aspects of their coaching philosophy.

**Figure 6:**
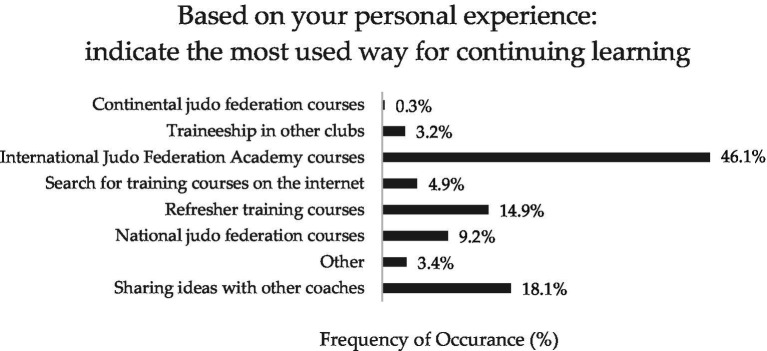
Answers’ percentages of the coaches’ perceived most suitable way for continuing learning.

Statistical analyses were conducted by means of the Jamovi software (version macOS, 2.6.22, x64.dmg) and the criterion for significance was set at a 0.05 alpha level. Data were organised in relation to the respondents’ demographic characteristics, as displayed in Table 2. Items from the Section 1 (age group, highest academic attainment, judo level, former competition level, judo education level, coaching experience, coaching older judo practitioners, salary for judo coaching) were considered as independent variables for inferential statistics. For all individual items of each section, descriptive statistics (mean and standard deviation or frequency of occurrence) were provided (Figures 1–6). The Shapiro–Wilk test was applied to verify the normality of the distribution. Because nominal data were gathered in this survey study and because data were not normally distributed, a Chi-Square test and non-parametric Mann–Whitney U and Kruskal-Wallis’ tests were conducted to account for potential between groups’ unequal sample sizes across the different independent variables. Regarding the Chi-Square test, to calculate the effect size, the Cramer *V* (*V*) test was performed. Values of *V* = 0.06–0.17 would refer to a small effect; *V* = 0.18–0.29 to a medium effect, and *V* > 0.30 would refer to a large effect size (Cramér, 1999). Rank biserial correlations (rank-biserial *r*) were performed to quantify the effect size for the Mann–Whitney *U* analysis. Values of rank-biserial *r* = <0.10 would refer to a very small effect; rank-biserial *r* = 0.10 to <0.30 to a small effect; rank-biserial *r* = 0.30 to <0.50 to a medium effect; rank-biserial *r* = ≥0.50 to a large effect size (Martin and Martinez, 2023). Additionally, we calculated Epsilon-squared (*ε*^2^) to assess the effect size for the Kruskal-Wallis’ tests, with thresholds classified as small (*ε*^2^ ≈ 0.01), medium (*ε*^2^ ≈ 0.06), and large (*ε*^2^ > 0.14) (Nunes et al., 2025). Note that, as this study was exploratory in nature, no correction for multiple comparisons was applied. Reported results should therefore be interpreted as preliminary and uncorrected, and future confirmatory studies may consider appropriate adjustment procedures.

**Table 2:** Socio-demographic characteristics of participants in the study.

Level	Count	Frequency of occurrence	
		(*n*)	(%)
Gender	Male	287	82
	Female	62	18
Age	≤39 years	108	31
	40–49 years	116	33
	≥50 years	125	36
Judo level	≥4 dan	161	46
	1–3 dan	188	54
Former elite	Yes	247	71
	No	102	29
Coach experience	11–20 years	137	39
	≥20 years	130	36
	1–10 years	82	24
Coach of intergenerational activities	Yes	253	73
	No	96	27
Volume of intergenerational training sessions	Low volume	169	48
	None	79	23
	High volume	101	29
Salary	Yes	202	58
	No	147	42
Academic level	High level	135	39
	Mid level	117	33
	Low level	97	28
Degree in sport sciences	Yes	144	41
	No	205	59

*n* = number.

## 3 Results

All the participants surveyed in this study were judo coaches involved in the 2023–2024 and 2024–2025 sport seasons of their respective judo clubs. Table 2 presents their socio-demographic characteristics, whereas Figures 1–6 report the mean and standard deviations of the items included in the six sections.

### 3.1 Factors influencing coaching philosophy

For coaching philosophy, the Chi-Square test indicated significant differences in relation to age group (*X*_2_ = 26.9; df = 14; *p* = 0.02; *V* = 0.196, medium effect), former experience as elite athletes (*X*_2_ = 16.8; df = 7; *p* = 0.019; *V* = 0.219, medium effect), coaching experience (*X*_2_ = 40.5; df = 14; *p* < 0.001; *V* = 0.241, medium effect), engagement in intergenerational training (*X*_2_ = 28.4; df = 14; *p* = 0.013; *V* = 0.202, medium effect), and degree in sport sciences (*X*_2_ = 25.9; df = 7; *p* < 0.001; *V* = 0.273, medium effect).

### 3.2 Continuing learning and professional engagement

The approach to continuing professional development was significantly associated with the judo level (*X*_2_ = 15.9; df = 7; *p* = 0.03; *V* = 0.214, medium effect) and degree in sport sciences (*X*_2_ = 14.1; df = 7; *p* = 0.049; *V* = 0.201, medium effect). The Chi-Square analysis indicated a significant difference between salary and coaching experience (*X*_2_ = 7.18; df = 2; *p* = 0.03; *V* = 0.143, small effect), judo level (*X*_2_ = 9.03; df = 1; *p* = 0.003; *V* = 0.161, small effect) and degree in sport sciences (*X*_2_ = 27.1; df = 1; *p* < 0.001; *V* = 0.279, medium effect), respectively. Specifically, 61% (*n* = 84) of the judo coaches with 11–20 years of coaching experience and 62% (*n* = 81) with ≥20 years, responded to receive a salary for their judo activity, unlike the most unexperienced group (1–10 years) in which only 45% (*n* = 37) reported an income out of their judo activity. Furthermore, 66.5% (*n* = 107) of the ≥4 dan level group declared receiving a salary, in contrast to 50.5% (*n* = 95) of the 1–3 dan level group. Furthermore, 74% (*n* = 107) of the coaches with a degree in sport sciences answered to get salary, while only 46% (*n* = 95) answered the same for the other group.

### 3.3 Gender differences in coaching perceptions

The Mann–Whitney *U* analysis indicated significant differences according to the gender of the participants in terms of ranking the relevance of inclusion (*U* = 7,476.0; *p* < 0.05; rank-biserial *r* = 0.160, small effect) and role model (*U* = 6,862.0; *p* = 0.004; rank-biserial *r* = 0.229) and perceived relevance of technical expert (*U* = 7,337.0; *p* = 0.023; rank-biserial *r* = 0.175). Male participants showed higher grade of inclusion relevance (mean ± SD = 4.2 ± 1.8 vs. 3.7 ± 1.8) and lower grade for role model (mean ± SD = 5.7 ± 1.6 vs. 6.1 ± 1.3) and perceived relevance of technical expert (mean ± SD = 4 ± 1.9 vs. 4.8 ± 1.7).

### 3.4 Age-related differences in coaching perceptions

Regarding the age of judo coaches, the Kruskal-Wallis’ test indicated significant differences for the perceived impact of lack of motivation and interest (*X*_2_ = 7.2; df = 2; *p* = 0.027; *ε*^2^ = 0.0207, medium effect) and social interactions (*X*_2_ = 6.56; df = 2; *p* = 0.038; *ε*^2^ = 0.0188, medium effect) and a significant difference on the opinion regarding the positive impactful of intergenerational mentorship (*X*_2_ = 6.17; df = 2; *p* = 0.046; *ε*^2^ = 0177, medium effect). For the impact of the lack of motivation and interest the pairwise comparisons revealed significant difference between coaches ≤39 years and ≥50 years (*p* = 0.019, mean ± SD = 5.2 ± 1.5 vs. 5.66 ± 1.5), while for the impact of social interactions a significant difference emerged between 40 and 49 years age category and ≥50 years (*p* = 0.031, mean ± SD = 5.54 ± 1.7 vs. 5.1 ± 1.7). Concerning the positive impactful of intergenerational mentorship, the pairwise comparisons found significant difference between ≤39 years and ≥50 years judo coaches (*p* = 0.034, mean ± SD = 4.36 ± 2 vs. 5.02 ± 1.9).

### 3.5 Influence of judo level

Considering the judo level the Mann–Whitney U test indicated significant differences for the relevance of the safety item (*U* = 13,242.0; *p* = 0.038; rank-biserial *r* = 0.125, small effect) and for the perceived impact of language and communication barriers (*U* = 13,242.0; *p* = 0.04; rank-biserial *r* = 0.126, small effect). In particular, the safety item was ranked higher by the coaches of 1–3 dan level compared to ≥4 dan (mean ± SD = 3.1 ± 1.8 vs. 2.8 ± 2.1), while for the impact of language and communication barriers the ≥4 dan level indicated higher rank than 1–3 dan (mean ± SD = 4.66 ± 1.8 vs. 4.24 ± 1.9).

### 3.6 Former elite experience

The Mann–Whitney U test indicated for the former elite condition (‘yes or no’) significant differences in the ranking of the role model (*U* = 10,888.0; *p* = 0.044; rank-biserial *r* = 0.136, small effect) and for the perceived impact of language and communication barriers (*U* = 10,897.0; *p* = 0.044; rank-biserial *r* = 0.135, small effect) and age differences (*U* = 10,742.0; *p* = 0.028; rank-biserial *r* = 0.147, small effect). For the role model ranking who was former elite judo athlete provided lower score compared to the group of no former elite (mean ± SD = 4 ± 1.9 vs. 4.45 ± 1.8), while vice versa were found for communication barriers (mean ± SD = 4.6 ± 1.8 vs. 4.1 ± 1.8) and age differences (mean ± SD = 4.4 ± 1.8 vs. 4 ± 1.7).

### 3.7 Coaching experience

According to judo coaches’ experience, the Kruskal-Wallis’ tests indicated significant differences in the perceived impact of mutual respect and empathy (*X*_2_ = 7.82; df = 2; *p* = 0.02; *ε*^2^ = 0.0225, medium effect) and between positive impactful of inclusive training sessions (*X*_2_ = 10.62; df = 2; *p* = 0.005; *ε*^2^ = 0.0305, medium effect), shared passion for sport and judo (*X*_2_ = 7.12; df = 2; *p* = 0.028; *ε*^2^ = 0.0205, medium effect) and supportive community and facilities (*X*_2_ = 10.12; df = 2; *p* = 0.006; *ε*^2^ = 0.0291, medium effect). The pairwise comparison demonstrated that the group with 1–10 years of experience significantly differed from their counterparts with 11–20 years and ≥20 years of experience, respectively: for the perceived impact of mutual respect and empathy, *p* = 0.041 and *p* = 0.029; for the impactful of inclusive training sessions, *p* = 0.027 and *p* = 0.005; for the impactful of shared passion for sport and judo *p* = 0.021 (only with ≥20 years group); and for the impactful of supportive community and facilities, *p* = 0.027 and *p* = 0.006.

### 3.8 Experience with intergenerational judo

The Mann–Whitney U analysis regarding previous experience in intergenerational judo activity revealed several significant differences in relation to: perceived relevance of adaptability specialist (*U* = 10,376.0; *p* = 0.032; rank-biserial *r* = 0.146, small effect), motivator (*U* = 10,167.0; *p* = 0.015; rank-biserial *r* = 0.163, small effect), role model (*U* = 10,276.0; *p* = 0.022; rank-biserial *r* = 0.154, small effect), technical expert (*U* = 9,824.0; *p* = 0.004; rank-biserial *r* = 0.191, small effect); perceived impact of environmental factors (*U* = 9,616.0; *p* = 0.002; rank-biserial *r* = 0.208, small effect), infrastructure challenges (*U* = 8,988.0; *p* = < 0.001; rank-biserial *r* = 0.260, small effect), lack of motivation and interest (*U* = 10,412.0; *p* = 0.034; rank-biserial *r* = 0.143, small effect); ranking of the relevance for the facilitator & organiser (*U* = 10,478.0; *p* = 0.045; rank-biserial *r* = 0.137, small effect), adaptability specialist (*U* = 10,270.0; *p* = 0.024; rank-biserial *r* = 0.154, small effect).

### 3.9 Volume of coaching activity

The Kruskal-Wallis’ tests regarding the volume of training also indicated significant differences in relation to the relevance ranking of community builder (*X*_2_ = 6.46; df = 2; *p* = 0.04; *ε*^2^ = 0.0186, medium effect), technical expert (*X*_2_ = 8.52; df = 2; *p* = 0.014; *ε*^2^ = 0.0245, medium effect), perceived impact of environmental factors (*X*_2_ = 7.03; df = 2; *p* = 0.03; *ε*^2^ = 0202, medium effect), and infrastructure challenges (*X*_2_ = 12.2; df = 2; *p* = 0.002; 0.0351, medium effect). The pairwise comparison revealed significant difference between none and high volume for the ranking of community builder (*p* = 0.04, mean ± SD = 4.8 ± 2 vs. 4.1 ± 2), between none and low volume (*p* = 0.03, mean ± SD = 3.2 ± 2 vs. 4 ± 2.3) and between none and high volume (*p* = 0.02, mean ± SD = 3.2 ± 2 vs. 4.2 ± 2.5) for the ranking of technical expert, between none and low volume for the environmental factor perceived impact (*p* = 0.013, mean ± SD = 4 ± 1.4 vs. 4.5 ± 1.6), between none and low volume (*p* = 0.003, mean ± SD = 4.1 ± 1.4 vs. 4.8 ± 1.6) and between none and high volume (*p* = 0.008, mean ± SD = 4.1 ± 1.4 vs. 4.8 ± 1.9) for the infrastructure challenges perceived impact.

### 3.10 Salary and professional status

The Mann–Whitney U test indicated significant differences between participants that received salary or not for the profession of judo coach in terms of perceived relevance of social interactions (*U* = 12,700.0; *p* = 0.016; rank-biserial *r* = 0.145, small effect), perceived impact of physical limitations (*U* = 12,469; *p* = 0.009; rank-biserial *r* = 0.160, small effect), positively impactful of shared passion for sport and judo (*U* = 12,977; *p* = 0.04; rank-biserial *r* = 0.126, small effect).

### 3.11 Academic education

Investigating on the academic level of participants, the Kruskal-Wallis’ test indicated only one significant difference for the perceived relevance of community builder (*X*_2_ = 8.36; df = 2; *p* = 0.015; *ε*^2^ = 0.0240, medium effect). The pairwise analysis revealed significant between mid-level and low level of academic studies (*p* = 0.014, mean ± SD = 5.4 ± 1.5 vs. 4.7 ± 1.7). Specifically, the Mann–Whitney U analysis indicated that having a degree in sport sciences or not was significantly different for the relevance ranking of collaboration item (*U* = 12,894.0; *p* = 0.04; rank-biserial *r* = 0.126, small effect) and adaptability specialist (*U* = 12,587.0; *p* = 0.02; rank-biserial *r* = 0.147, small effect), for the impactful ranking of mutual respect and empathy (*U* = 12,946; *p* = 0.04; rank-biserial *r* = 0.123, small effect) and passion for sport and judo (*U* = 12,554; *p* = 0.02; rank-biserial *r* = 0.149, small effect). Coaches that had degree in sport sciences presented lower scores compared to their counterparts for the relevance ranking of collaboration (mean ± SD = 3.51 ± 1.5 vs. 3.9 ± 1.7) and adaptability specialist (mean ± SD = 3.58 ± 2.3 vs. 4.1 ± 2.2) and greater score for the impactful ranking of shared passion for sport and judo (mean ± SD = 4.5 ± 2.2 vs. 3.9 ± 2.1) and mutual respect and empathy (mean ± SD = 5.7 ± 1.4 vs. 5.5 ± 1.4).

## 4 Discussion

Aiming to ground solid foundations for future educational and sport strategies of intergenerational judo activities, this study uncovered relevant opinions, knowledge, and experiences of international judo coaches. The achieved response rate of 16% can be considered acceptable within the context of online surveys in the sport sector, where participation is often voluntary and respondents are typically practitioners with demanding schedules. Similar or lower rates have been reported in studies targeting coaches, athletes, or sport organisations, reflecting common challenges in reaching this population (DiSanti et al., 2019). Moreover, declining response rates have been observed across online research more broadly (Baruch and Holtom, 2008), making the present rate consistent with trends in sport and exercise science surveys that rely on professional networks and email distribution.

Results from the tailored survey reveal a high perceived relevance of all intergenerational key characteristics selected from the literature (Ciaccioni et al., 2024b), with safety and skill exchange emerging as the most important according to the participants’ evaluations (Figure 1). In line with the expectation that an intergenerational judo coach should inspire, guide, and transmit technical skills (Ciaccioni et al., 2024b), the functions of motivator, role model, and technical expert appear to be slightly valued (Figure 2). Moreover, whilst all facilitators are seen as potential means of support and assistance without predominance, the judo coaches indicated physical limitations and lack of motivation and interest as the most influential barriers (Figures 3, 4).

However, coaches’ life experiences impact their daily practice and determine relevant changes in their coaching principles over time (Perazzetti et al., 2023; Barrenetxea-Garcia et al., 2024). Therefore, to guide decision-making overcoming practical problems and favouring consistency in coaching, both vocational and professional sport coaches need to develop their own coaching philosophy (Lyle, 2002; Cassidy et al., 2009). To deepen our knowledge on this aspect, the judo coaches were asked to rate the most influential factors for their coaching philosophy. Experience and mentorship seem to play a crucial role since the highest-rated responses were ‘years of continuous practice as a judo coach’ (32.7%), ‘own experience as a judo athlete’ (19.5%), and ‘having other coaches as mentors’ (19.2%), while formal education appeared less influential (i.e., ‘having a degree in sport sciences’ rated by only 5.7% of participants). In line with previous studies emphasizing the importance of promoting educational initiatives and well-structured planning for coach development (Ciampolini et al., 2020), continuous education seems to assume a crucial role with 46.1% of participants recognizing the value of the courses offered by the IJFA.

This finding suggests that coaching philosophies in judo are shaped far more by practical experience and mentoring than by formal education. Such evidence may reveal a possible gap between the academic content delivered in coach education programmes and the real needs or contexts of judo coaches. It raises relevant questions: are existing educational systems struggling to connect with coaches’ practical realities, or are barriers limiting coaches’ access to meaningful and applied learning opportunities? This contrast may reflect a deeper cultural tension between the traditional, apprenticeship-based pedagogy typical of martial arts (master-disciple learning) and the modern, science-driven model of sports education. While the traditional approach preserves authenticity and lineage, it may also engender scepticism toward formal instruction. Therefore, future training initiatives for judo coaches should aim to balance tradition and innovation, being, as the name ‘judo’ implies, flexible and adaptable (‘ju’), while integrating scientific evidence in a way that respects and complements the heritage of martial arts (Ciaccioni et al., 2025; Lee et al., 2025).

To note, judo coaching philosophy showed to be associated with many factors including: (i) age, confirming that with advancing years the coaches accrue experience, which may influence their approach to training attitudes and styles (Lara-Bercial and Mallett, 2016); (ii) being a former elite athlete, emphasizing how coaching beliefs are influenced by individual competitive experience (Werthner and Trudel, 2006); (iii) a degree in sport sciences, supporting the notion that formal education offers a structured basis for coaching approaches (Nash et al., 2017).

The relationship between lifelong learning, holding a degree in sport sciences, and judo level highlights the critical role of both academic education and technical proficiency in a judo coach’s development. In fact, coaches with a higher level (≥4 dan level) demonstrate a greater propensity to pursue further education and stay updated with the latest coaching methodology (Ciaccioni et al., 2024c). Significant relationships also emerged between salary, coaching experience, judo level, and educational attainment with more experienced coaches and those holding higher dan ranks being more likely to receive a salary, speculating that remuneration is often linked to expertise and reputation within the field.

From a broader perspective, this result may highlight the difference between two professional realities within the judo community: the ‘professional coach,’ often employed and working full-time, and the ‘volunteer or amateur coach.’ The professional coach, being more immersed in everyday practice, tends to adopt a holistic vision of intergenerational programmes (considering aspects such as facilities, community, and participant motivation) while the volunteer coach may focus primarily on technical and pedagogical dimensions. This distinction suggests that educational initiatives should consider the specific profiles and working conditions of different types of coaches. Importantly, salary and workload do not merely represent compensation but act as catalysts for the development of broader pedagogical and managerial skills, ultimately enhancing the quality and sustainability of intergenerational judo programmes.

Recent judo-based research explored various gender differences related to injury risk, physiological parameters, psychological characteristics and performance metrics (Marković et al., 2018; Blanco-García et al., 2021; Prados-Barbero et al., 2024; Türkçapar and Abdirahmanova, 2024). In the present study, male and female participants differed significantly in their views on inclusion, role models, and technical expertise in judo coaching with men placing greater emphasis on inclusion, while women prioritizing technical competence and the coach’s role as a model. These differences may reflect broader gender-related perspectives, with men potentially focusing more on structural aspects such as accessibility and participation, whereas women may place greater value on relational and competence-based qualities, possibly due to the historically greater challenges they face in accessing high-quality coaching and role models in sport contexts (Miarka et al., 2011; Da Silva et al., 2023).

Beyond these observed differences, such patterns may also represent adaptive strategies within a historically male-dominated martial arts environment. Female coaches, striving for legitimacy, may prioritize technical excellence and ethical conduct (being a role model), whereas male coaches, already operating from a position of established authority, may have the freedom to focus more broadly on inclusion and structure. This interpretation suggests that gender-related differences in perception might not merely reflect preferences but adaptive responses to systemic dynamics within judo. Therefore, policies and educational programmes should consider gender and power relations in order to foster more equitable and effective learning and coaching environments.

Moreover, as coaches age, their priorities appear to shift. With an accumulated experience, they may focus increasingly on tutoring and inspiring athletes, whereas mid-career coaches maintain a stronger emphasis on fostering social relationships within the coaching environment. In fact, compared to younger counterparts (≤39 years), older coaches (≥50 years) placed greater value on intergenerational mentorship and demonstrated heightened awareness for the negative impact of lack of motivation on athletes, but emphasized social interactions less than coaches aged between 40 and 49. These results reflect previous studies showing that coaches, especially those working with older adults, recognize the need for innovative methods to improve athletes’ experiences (Ciaccioni et al., 2024a). This includes creating environments and educational opportunities where safety, enjoyment, social bonds, and learning are all connected to better support athletes’ development, morality and personality formation as well as wellbeing (Kolb, 2014; Hryban and Filina, 2022). Whilst coaches with lower rank (1–3 dan) prioritized safety more strongly, their higher rank (≥4 dan) counterparts expressed greater concern about communication and language barriers, likely reflecting their broader involvement in international context. To note, despite national variations in the 206 member Countries included in the International Judo Federation, worldwide dan promotions combine randori (i.e., free practice combat exercise), kata (i.e., formally codified practice exercise), and either competitive achievements or technical demonstrations, with increasing requirements for higher dan ranks (Calmet et al., 2024).

Concerning athletic career-related differences in perceived key-aspects of judo intergenerational activities, in this study former elite coaches attributed less importance to the coach’s role model function compared to their non-elite counterparts. Actually, the literature highlights that high-level judo coaches’ perspectives are shaped by their combined experiences as athletes and coaches, relying on both traditional and modern methodologies as well as on a complex set of personal believes on physical, technical, tactical and psychological elements needed for high-level performance achievements (Santos et al., 2015).

Another important aspect considered in the present study is the influence of the number of weekly hours that coaches dedicate to intergenerational judo activities. Coaches with higher weekly coaching volumes place greater importance on their roles as technical expert and community builder, suggesting that frequent and active involvement in intergenerational coaching sharpens their recognition of the need for technical competence and the ability to foster community dynamics. Moreover, they also showed a great awareness of environmental and infrastructure-related barriers (e.g., facility limitations or unfavourable training conditions). Conversely, coaches with low or no weekly coaching activity appeared less focused on these aspects, likely due to their limited practical exposure to the specific challenges encountered during regular intergenerational practice.

Salary represents another critical factor for judo athletes, coaches, and referees as it supports their professionalization, sustains long-term engagement, and acknowledges their expertise and contributions to the development of the sport (Kim and Shin, 2014; Perondi et al., 2022; Ciaccioni et al., 2024c). Therefore, employment conditions were also explored to understand the related influence on the coaches’ believes. Indeed, a professional engagement with a salary seems to allow coaches to attribute a great importance to social interactions, to demonstrate a high awareness of the physical limitations of the practitioners, and to place a relevant value to the shared passion for sport engagement and judo practice. This pattern suggests that employed coaches may develop a broad perspective on their coaching role in establishing and nurturing interpersonal relationships, recognizing both the physical and psychosocial dimensions of an effective intergenerational practice (Ciaccioni et al., 2024c). Also, the educational background appears to shape coaches’ perceptions of key competencies and values (Junior et al., 2024). Within intergenerational judo activities, coaches with a mid-level academic background might consider community building very important, suggesting that education could enhance their understanding of aspects relevant to community dynamics. Furthermore, it could be possible to speculate that coaches without a specific higher education in sport sciences emphasize collaboration and adaptability, which reflect their reliance on practical skills, whereas coaches with a higher education in other areas might prioritize mutual respect and passion for sport, being primarily influenced by their past experience as judo athletes (Guidotti et al., 2023a; Guidotti et al., 2023b; Ciaccioni et al., 2024c).

Despite its valuable findings, this study has several limitations that should be noted. The international approach of this work could limit the generalizability of the findings to specific geographic or cultural contexts. Furthermore, the survey applied in the present study was based on quantitative data with only one open-ended question, which allowed to collect information on a wide range of topics but reduced the possibility to deepen our understanding on the reasons behind responses. Finally, the survey involved only coaches, without taking into consideration the practitioners’ or athletes’ point of view. Therefore, future studies should consider the adoption of mixed methods approaches, including in-depth interviews or focus groups, to better understand the complexity of coaches’ experiences. Also, the findings could benefit from the insights of the young and older practitioners, which could ensure a thorough understanding of the programme effectiveness. Also, longitudinal studies are recommended to assess how educational interventions and intergenerational programmes impact coaches’ philosophies and practices over time. For example, intervention studies testing specific educational modules or training strategies could further validate best practices for promoting inclusive, sustainable, and effective intergenerational judo programmes across different contexts. Moreover, as this study represents the first application of the proposed survey tool, a full psychometric validation was beyond its scope. Future research should therefore aim to further validate the instrument’s structure, reliability, and cross-cultural applicability. In addition, future investigations should explore how intergenerational coaching philosophies evolve through time, and how specific educational or mentoring interventions can foster positive changes in coaching attitudes and behaviours. Mixed-method or longitudinal designs would be particularly useful to capture these dynamic processes and to provide richer, evidence-based recommendations for coach education and policy development.

## 5 Conclusion

The present study adopted ecologically valid settings, considering real-world scenarios (Pernigoni et al., 2024) that can inform the development of specific training strategies for intergenerational judo (Ciaccioni et al., 2024b). These findings offered valuable suggestions regarding the opinion of judo coaches on intergenerational judo activity. By identifying coaches’ perceptions on key characteristics, roles, barriers, and facilitators of intergenerational judo programmes, this work provided a framework for developing tailored educational strategies that foster cooperation between younger and older generations in judo and stimulated further research in this area. In particular, the present results highlight the importance of integrating elements such as safety, mentorship, social interactions, and mutual respect, which are crucial for enhancing the effectiveness and inclusivity of intergenerational judo programmes, contributing to the goal of promoting active ageing and social cohesion between generations through sport.

## Data Availability

The raw data supporting the conclusions of this article will be made available by the authors without undue reservation.
